# Supplementary material for: The causal role of circulating inflammatory markers in osteoporosis: a bidirectional Mendelian randomized study
Source: Front Immunol. 2024 Jul 18;15:1412298. doi: 10.3389/fimmu.2024.1412298 (PMC11291241; doi:10.3389/fimmu.2024.1412298)
Supplement: Supplementary file 1 [file DataSheet_1.zip › supplementary material/CIM-OSTEOPOROSIS /GCST90274838.csv_leave_one_out.pdf]

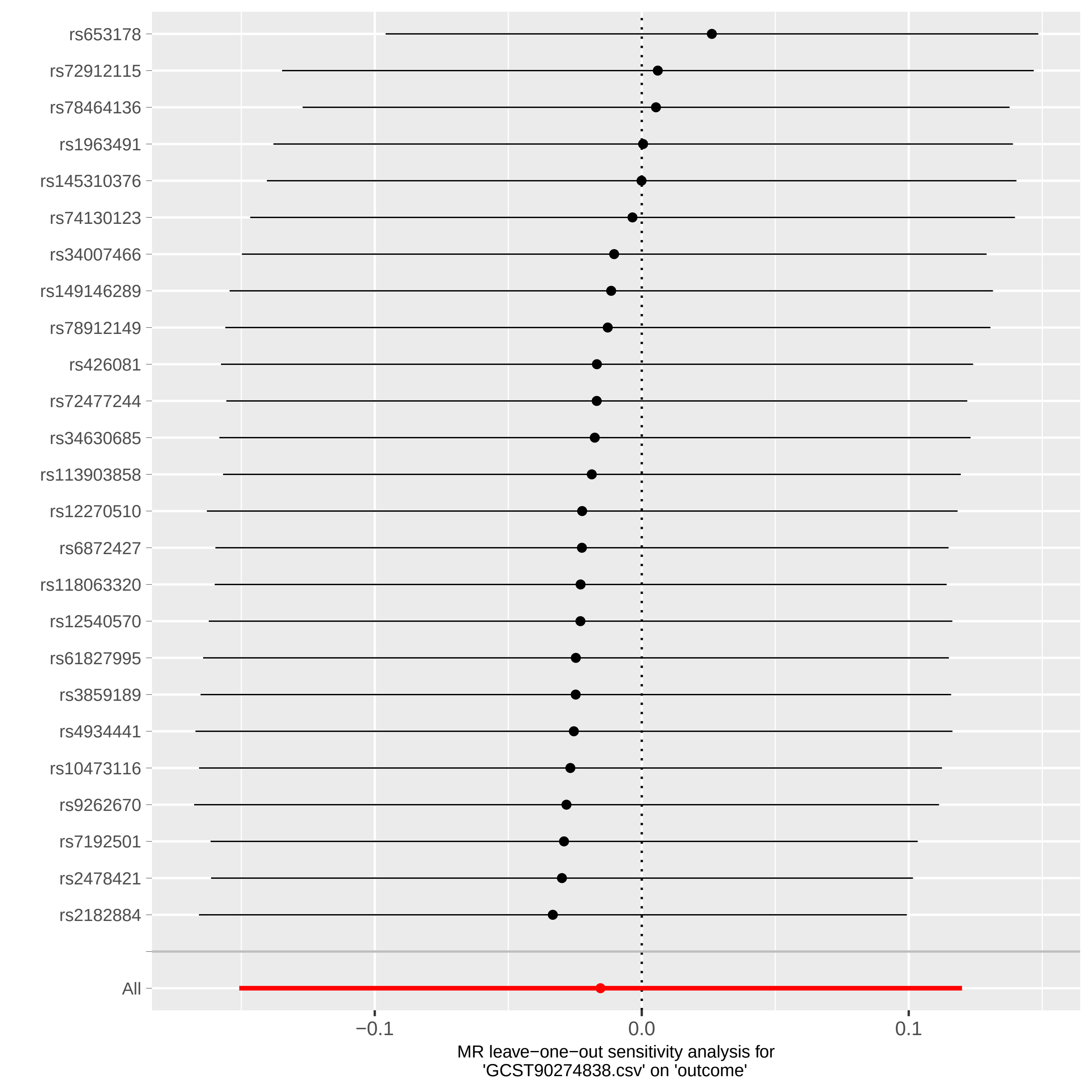
rs653178
rs72912115
rs78464136
rs1963491
rs145310376
rs74130123
rs34007466
rs149146289
rs78912149
rs426081
rs72477244
rs34630685
rs113903858
rs12270510
rs6872427
rs118063320
rs12540570
rs61827995
rs3859189
rs4934441
rs10473116
rs9262670
rs7192501
rs2478421
rs2182884
All
−0.1
0.0
0.1
MR leave−one−out sensitivity analysis for
'GCST90274838.csv' on 'outcome'
